# Adaptive simulations, towards interactive protein-ligand modeling

**DOI:** 10.1038/s41598-017-08445-5

**Published:** 2017-08-16

**Authors:** Daniel Lecina, Joan F. Gilabert, Victor Guallar

**Affiliations:** 1 Barcelona Supercomputing Center (BSC), Jordi Girona 29, E-08034 Barcelona, Spain; 2 ICREA, Passeig Lluís Companys 23, E-08010 Barcelona, Spain

## Abstract

Modeling the dynamic nature of protein-ligand binding with atomistic simulations is one of the main challenges in computational biophysics, with important implications in the drug design process. Although in the past few years hardware and software advances have significantly revamped the use of molecular simulations, we still lack a fast and accurate *ab initio* description of the binding mechanism in complex systems, available only for up-to-date techniques and requiring several hours or days of heavy computation. Such delay is one of the main limiting factors for a larger penetration of protein dynamics modeling in the pharmaceutical industry. Here we present a game-changing technology, opening up the way for fast reliable simulations of protein dynamics by combining an adaptive reinforcement learning procedure with Monte Carlo sampling in the frame of modern multi-core computational resources. We show remarkable performance in mapping the protein-ligand energy landscape, being able to reproduce the full binding mechanism in less than half an hour, or the active site induced fit in less than 5 minutes. We exemplify our method by studying diverse complex targets, including nuclear hormone receptors and GPCRs, demonstrating the potential of using the new adaptive technique in screening and lead optimization studies.

## Introduction

Accurately describing protein-ligand binding at a molecular level is one of the major challenges in biophysics, with important implications in applied and basic research in, for example, drug design and enzyme engineering. In order to achieve such a detailed knowledge, computer simulations and, in particular, molecular *in silico* tools are becoming increasingly popular^1, 2^. A clear trend, for example, is seen in the drug design industry: Sanofi signed a $120 M deal with Schrödinger, a molecular modeling software company, in 2015. Similarly, Nimbus sold for $1,200 M its therapeutic liver program (a computationally designed Acetyl-CoA Carboxylase inhibitor) in 2016. Clearly, breakthrough technologies in molecular modeling have great potential in the pharmaceutical and biotechnology fields.

Two main reasons are behind the revamp of molecular modeling: software and hardware developments, the combination of these two aspects providing a striking level of accuracy in predicting protein-ligand interactions^1, 3, 4^. A remarkable example constitutes the seminal work of Shaw’s group, where a thorough optimization of hardware and software allowed a complete *ab initio* molecular dynamics (MD) study on a kinase protein^5^, demonstrating that computational techniques are capable of predicting the protein-ligand binding pose and, importantly, to distinguish it from less stable arrangements by using atomic force fields. Similar efforts have been reported using accelerated MD through the use of graphic processing units (GPUs)^6^, metadynamics^7^, replica exchange^8^, etc. Moreover, these advances in sampling capabilities, when combined with an optimized force field for ligands, introduced significant improvements in ranking relative binding free energies^9^.

Despite these achievements, accurate (dynamical) modelling still requires several hours or days of dedicated heavy computation, being such a delay one of the main limiting factors for a larger penetration of these techniques in industrial applications. Moreover, this computational cost severely limits examining the binding mechanism of complex cases, as seen recently in another study from Shaw’s group on GPCRs^10^. From a technical point, the conformational space has many degrees of freedom, and simulations often exhibit metastability: competing interactions result in a rugged energy landscape that obstructs the search, oversampling some regions whereas undersampling others^11, 12^. In MD techniques, where the exploration is driven by numerically integrating Newton’s equations of motion, acceleration and biasing techniques aim at bypassing the highly correlated conformations in subsequent iterations^13^. In Monte Carlo (MC) algorithms, another main stream sampling method, stochastic proposals can, in theory, traverse the energy landscape more efficiently, but their performance is often hindered by the difficulty of generating uncorrelated protein-ligand poses with good acceptance probability^14, 15^. The Protein Energy Landscape Exploration (PELE) program^16^ addresses the problem by making use of protein structure prediction algorithms, which introduces larger conformational changes^17^ and, importantly, allows mapping complex protein-ligand binding mechanisms^18–20^. This technique, for example, has been underlined as an impressive accomplishment in the last Community Structure-Activity Resource (CSAR) blind competition^21^. Nonetheless, PELE simulations still show some degree of metastability, requiring several hours for solving the binding mechanism in complex systems, restricting its use in a drug design screening setup. For introducing large impact, we should aim for fast (minutes) and accurate simulations, allowing a drug design team to obtain accurate protein-ligand structures interactively, opening the possibility to combine their knowledge and expert intuition with *in silico* techniques on-the-fly. In this work, we present such a breakthrough tool: Adaptive-PELE, a combination of PELE with an adaptive reinforcement learning procedure.

Of particular interest in our study are iterative methods making use of short simulations and deciding on-the-fly the most interesting regions to sample, such as adaptive sampling^8^, weighted ensemble^22^, the adaptive seeding method^23^, or the FAST^24^ technique. The latter method rewrites the conformational exploration in terms of the well-studied multi-armed bandit (MAB) problem^25^, taking advantage of the gradient existing in measurables, such as the solvent-accessible surface area (SASA) or some energy components. We understand the ligand-protein exploration as an exploration-exploitation dilemma, since the phase space is highly dimensional and sufficient sampling of relevant regions, not only of a few metastable states, is necessary for an accurate characterization. The exploration is a learning process where we acquire knowledge of the energy landscape as the simulation progresses, and we decide to focus on the most rewarding regions. We serve of the MAB as a theoretical framework, since it has been successfully applied in a wide range of problems such as protein folding^24^, on-line advertising or news recommendation^26^.

**Figure 1:**
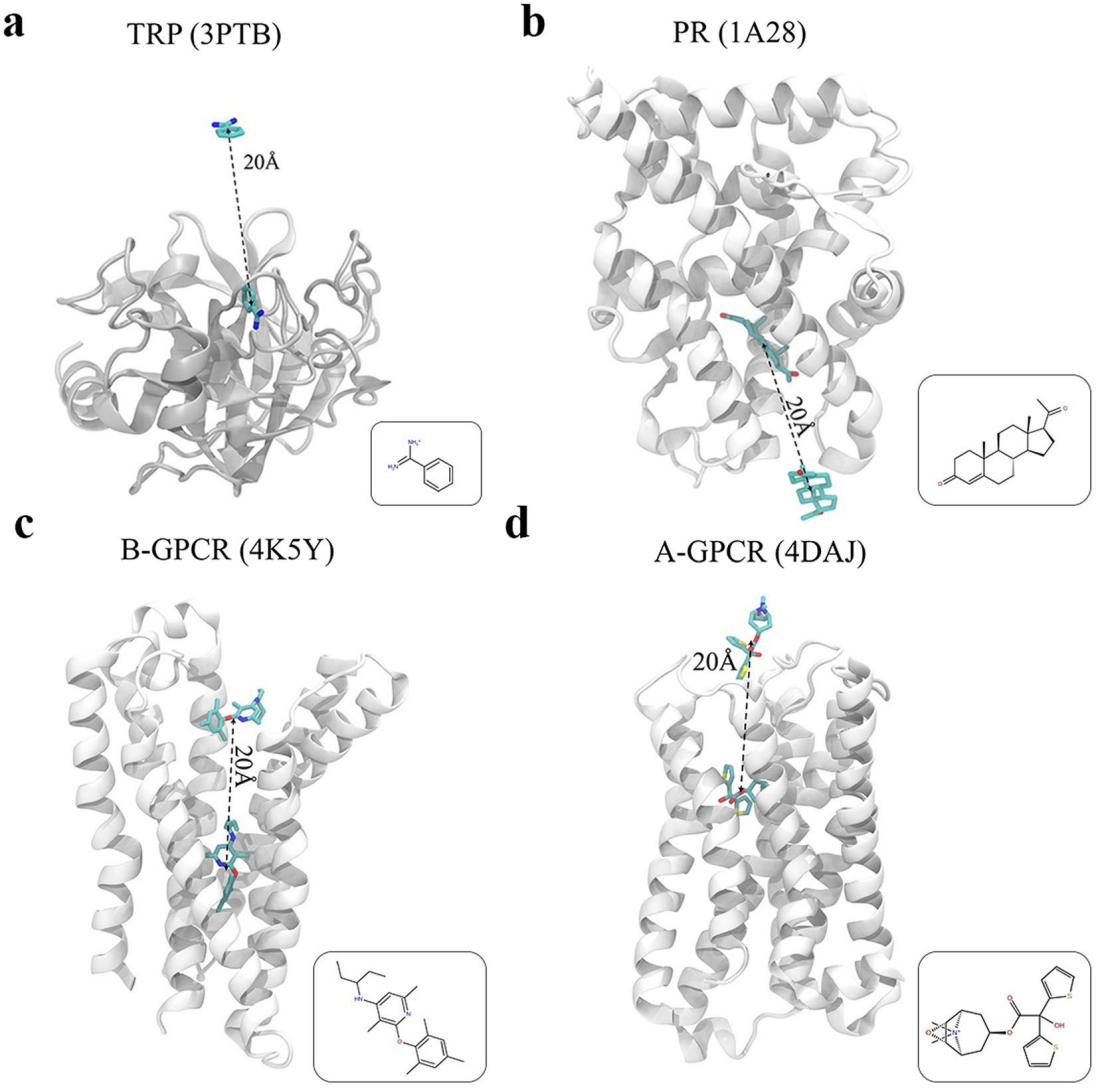
Protein-ligand complexes studied. (**a**) Trypsin with benzamidine as a ligand (TRP, PDB ID: 3PTB). (**b**) Progesterone nuclear hormone receptor with progesterone as a ligand (PR, PDB ID: 1A28). (**c**) Corticotropin-releasing factor GPCR with CP-376395 as a ligand (B-GPCR, PDB ID: 4KY5). (**d**) M3 muscarinic acetylcholine GPCR with tiotropium as a ligand (A-GPCR, PDB ID: 4DAJ). The initial structures for the protein-ligand exploration, with the ligand ~20 Å away from the binding site, are shown. The square inset in each panel depicts a 2D scheme of each ligand.

Adaptive-PELE is based on an iterative procedure where each iteration, referred as an epoch, involves three different steps: exploration, clustering and spawning (or seeding). Its landscape exploration capabilities, confronted with standard PELE executions (non-adaptive trajectories), are shown in four different protein-ligand complexes (Fig. 1): i) the trypsin—benzamidine (TRP system); ii) a progesterone nuclear hormone receptor with its endogenous ligand (PR system), iii) the M3 muscarinic acetylcholine class A G-protein coupled receptor (GPCR) with an inverse agonist (A-GPCR system); iv) the corticotropin-releasing factor, a class B GPCR with an antagonist ligand (B-GPCR system). Our results demonstrate that the new adaptive technique is capable of mapping the binding energy landscape for complex systems in less than half an hour, or the active site induced fit process in less than 5 minutes.

## Results

### Energy landscape exploration

**Figure 2:**
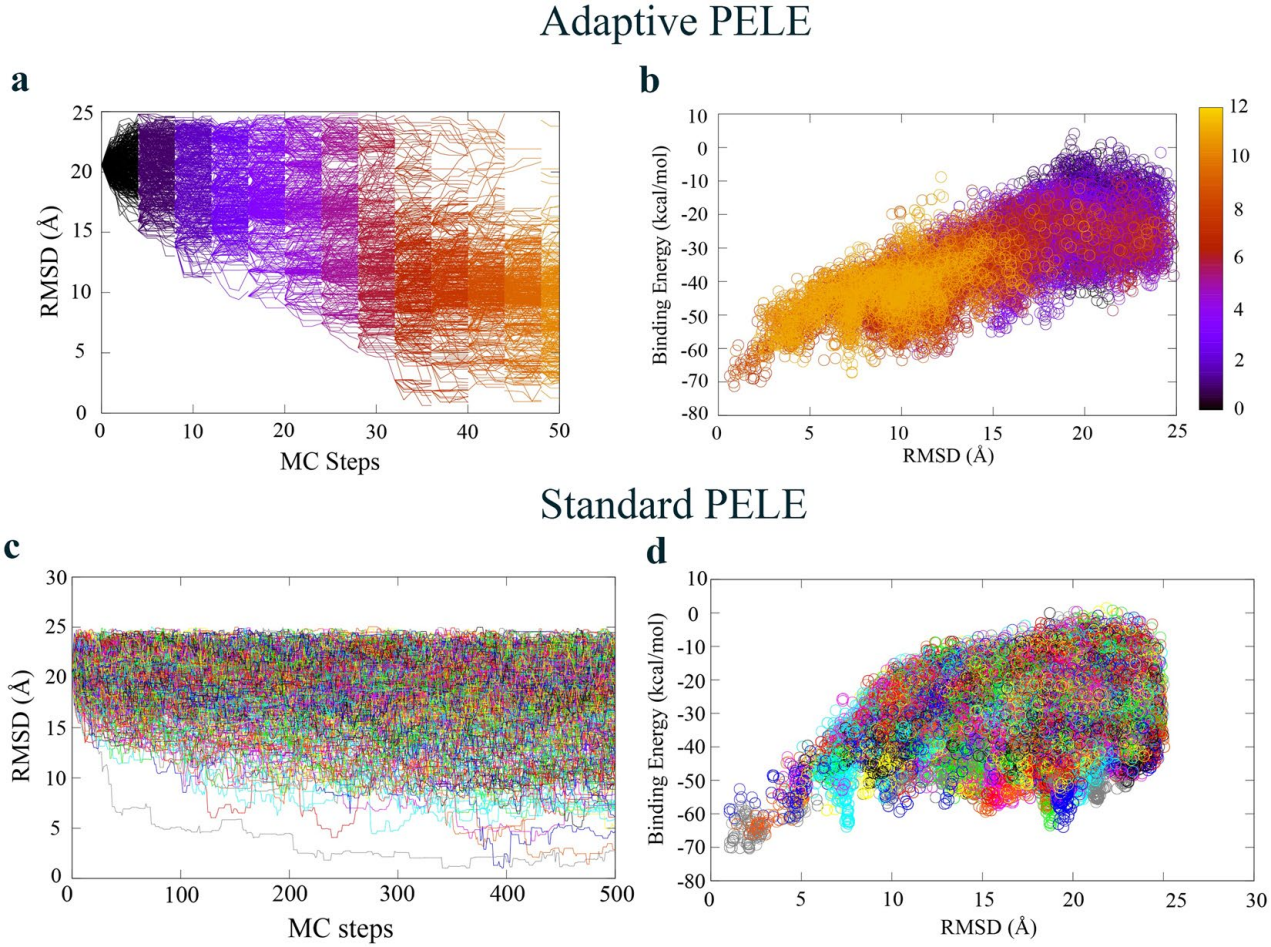
Energy landscape exploration of B-GPCR with 512 different explorers. (**a,b**) The RMSD variation along MC steps and the binding energy against the RMSD for the adaptive results. Each color code corresponds to a different epoch number, for a total of 12 adaptive iterations. (**c,d**) Analogous plots for the standard executions. Each color corresponds to a different trajectory (performed in a different computing core). Notice the change in scale in the X-axis between (**a**) and (**c**).

We first show the protein-ligand energy landscape exploration capabilities of Adaptive-PELE and compare them to that of a standard (non-adaptive) procedure. The evolution of the ligand root mean square deviation (RMSD) to the native bound structure along the simulation (MC steps), and the protein-ligand binding energy against the same ligand RMSD is shown in Fig. 2. We plot here the results for the B-GPCR system, using 512 trajectories (each trajectory runs in a computing core), but equivalent figures for the remaining systems are shown in the Supplementary Information. As seen in the RMSD evolution plots, both the adaptive (Fig. 2a) and standard (Fig. 2c) PELE methods succeed in sampling native-like conformations, with RMSD values ~1 Å; analogous results are seen for all other systems (Supplementary Figs. 2 to 4). We should emphasize that the initial starting pose for the ligand is significantly away from the binding site (~20 Å, Fig. 1) and that there is no bias in the search: no information from the bound pose is used but for plotting purposes. Such a non-biased sampling performance, for example, has not been successful for MD techniques in complex systems such as the A-GPCR, only seeing the binding to an extracellular site vestibule, approximately at 12 Å from the bound structure, when using 16 μs of standard MD^10^ or 1 μs of accelerated MD^27^.

As we can see in Fig. 2a and b, the first phase of the adaptive simulation is devoted to explore the bulk and the vicinity of the initial pose. Significantly, as the adaptive epochs evolve few simulations enter deeper into the cavity, getting into an unexplored region. The MAB strategy uses this information to spawn several explorers there, increasing the possibilities of finding new unexplored areas. Towards the end of the sampling, we observe an almost complete shift of the explorers towards the binding site region. The standard PELE technique, however, keeps exploring the outer regions (Fig. 2c and d), with minimal excursions into the binding site, resulting in a much less efficient exploration (see below for a thorough comparison). A nice additional feature is that the exploration moves away from regions once they are sufficiently known, avoiding metastability. For example, the binding pose is found at around step 30, and the sampling is only kept there two more epochs, when exploration efforts are moved to more rewarding areas.

A noteworthy common aspect in both techniques is that we can easily identify the native-like pose using the binding energy. The potential of using PELE’s binding energy, an all atom OPLS2005 protein-ligand interaction energy with an implicit solvent model, in pose discrimination was already shown in our initial induced-fit benchmark study^28^, being also the basis for our recent success in the CSAR blind competition. While this energy does not correlate with absolute experimental affinities (nor allows us to compare different ligands), it is very useful for pose discrimination; similar observations have emerged when using MD^5^. Importantly, introducing the adaptive procedure improves the binding energy landscape funnel shape, avoiding an unbalanced exploration of metastable regions, which eliminates the severe optimization on the energy by constantly minimizing over and over the same minimum. This can be seen, for example, when comparing the difference in “binding peaks” at 7.5 and 20 Å in Fig. 2b and d.

### Binding event observation - Binding time

The ligand finds native-like poses in ~35 MC steps when using the new adaptive approach (Fig. 2a), the independent PELE simulation requiring approximately 10 more times, ~350 steps (Fig. 2c). While standard PELE already represents a significant advance over other sampling techniques (microsecond MD simulations with the Anton computer, for example, could not observe a binding event for A-GPCR^10^), the adaptive scheme introduces a remarkable speed up. As a rule of thumb, each MC PELE step takes around 45 seconds on a SandyBridge-EP 2.6 GHz computing core, and therefore, in this particular simulation the bound native structure can be predicted in under 30 minutes using the adaptive approach.

To quantitatively assess our new algorithm’s performance, we estimated the binding times by averaging over ten separate runs, considering that a binding event occurred when the ligand RMSD with the native bound structure was less than 2.5 Å. In addition, we checked the scalability by using an increasing number of trajectories (computing cores), from 32 to 1024, summing up to a total computing time of a quarter of million CPU hours. Moreover, different MAB strategies (see the Methods section) were used for the adaptive simulations, including the inversely proportional and *ε*-greedy, guiding the exploration with two metrics: the protein-ligand interaction energy, where the native structure does not need to be known, and the ligand RMSD to the native, a biased strategy that allows us to estimate a lower bound for the binding time. Notice that when using a small number of explorers some standard PELE simulations did not produce binding events in 3000 MC steps. In those cases, we assigned the binding time to 3000 steps in order to set a lower bound for the comparison.

**Figure 3:**
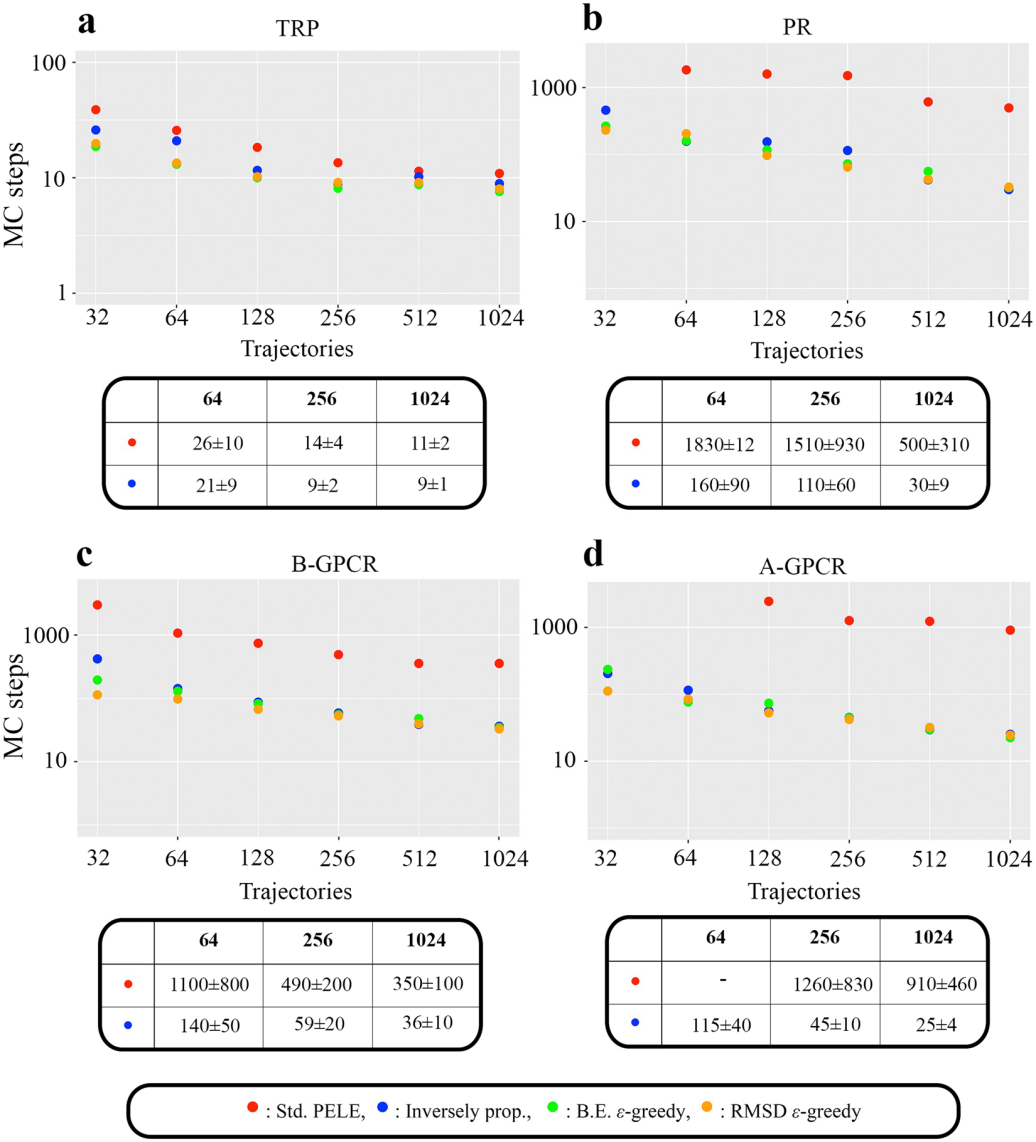
Binding times for all systems and MC techniques. (**a**) Number of steps for observing a binding event against the number of trajectories (processors) for the TRP system, using the standard PELE (in red) and the adaptive-PELE with the inversely proportional (in blue) and the *ε*-greedy guided strategies with binding energy (in green) and RMSD (in orange). Actual data (MC steps) with their standard deviation for three different sets of processors is shown at the bottom table inset for the standard PELE and the inversely proportional adaptive-PELE methods. (**b–d**) Analogous plots for PR, B-GPRC, and A-GPCR. A complete list of all data is shown in Supplementary Information.

We observe that in general the binding time decreases with the number of processors for all systems and methods (Fig. 3). In TRP, however, we approach a plateau for 256 processors; adding up more explorers only yields minor improvements. TRP is a relatively rigid protein not requiring structural rearrangements to bind benzamidine, and using 256 processors we almost reach the minimum possible binding time, given the ligand translation range per MC step and the starting position. In the remaining three (more difficult) systems, however, the binding time keeps decreasing in the whole range, since we need a more exhaustive protein sampling, and ligand movements need to couple to protein rearrangements.

In agreement with the difficulties seen in MD simulations, the exploration in A-GPCR is especially poor for the standard PELE approach, not seeing a significant number of binding events with less than 128 trajectories. It is quite remarkable that by introducing the adaptive sampling we find the correct binding mode using 32 cores in only ~3 hours of simulation. The overall speed up achieved by adaptive-PELE for this system is approximately 40 times in the studied number of processors range, being at least one order of magnitude in the other two complex systems, PR and B-GPCR. As expected, TRP has the least speed up gain, since it is the least computationally demanding example. Importantly, for all studied systems the adaptive technique is capable of providing native-like poses in less than half an hour when a large number of computing cores is provided, a significant achievement.

Interestingly, the different MAB strategies perform quite similarly. Guiding the seeding with the protein-ligand binding energy does not require previous knowledge of the binding site and, as emphasized above, it correlates nicely with the native-like pose (although it has been reported that sometimes the SASA has been shown to perform better^29^). In addition, if one has available the bound crystal structure, one can use the RMSD to guide the binding, which serves as an estimation of the binding time limit that we could achieve; a similar strategy could be obtained by simply knowing the binding site and using its distance to the ligand’s center of mass to guide the spawning. Surprisingly, when increasing the number of processors all these strategies yield similar results as our default option, the inversely proportional strategy, which seems to indicate that the choice of the reward function depending on the number of contacts (see Methods section) makes quite an optimal seeding.

### Mechanistic studies: protein conformation exploration

While we have shown that adaptive-PELE can provide native-like poses in complex systems in a fast manner, it is important to show that it also provides the proper binding mechanism. We show here the analysis for two of the more difficult systems, PR and A-GPCR.

**Figure 4:**
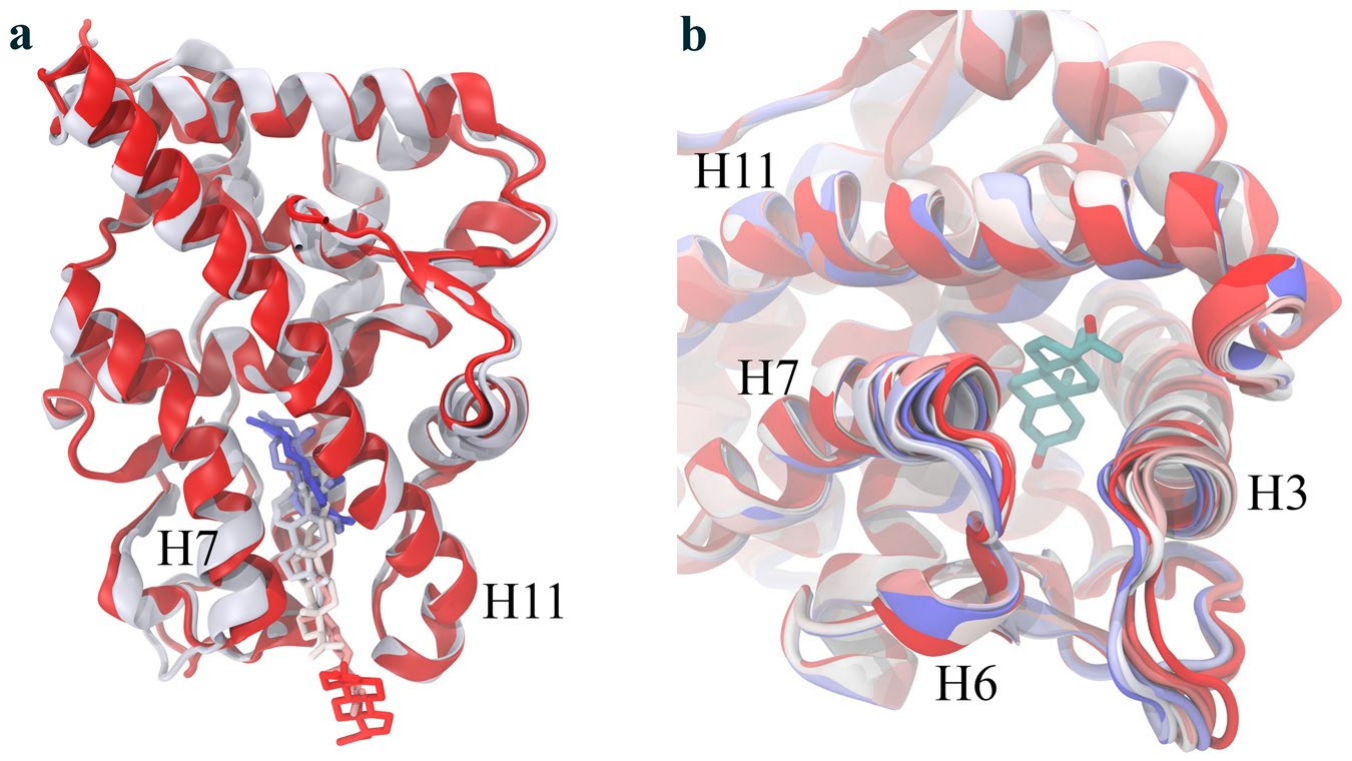
PR binding mechanism. Two different views of the ligand entrance and the plasticity upon progesterone binding in PR. **(a)** Different ligand snapshots along the binding with two protein structures highlighting the initial closed (red cartoon) and intermediate open states (white cartoon). **(b)** A closer zoom at the entrance region with the ligand shown in the native bound structure; same color-coding as in the (a) panel but for the ligand (shown with atom element colors).

PR. Recent crystallographic and computational studies in NHRs have underlined the conformational changes necessary for ligand delivery at the entry site: helices 3, 6, 7 and 11, along with the loops linked to them^19, 30^; with respect to this region, NHRs seem to adopt an open and a closed structure coupled to the ligand’s entrance. The PR receptor, in particular, has the largest plasticity in this region, as shown in the PCA analysis on all available NHRs bound crystal structures^30^. Such conformational change is well captured by the adaptive technique. As seen in Fig. 4, the protein starts in the closed conformation (shown in red) and achieves its largest opening when the ligand starts entering the cavity from the peripheral binding site (shown in white), to progressively close again towards the native pose as it gets deemed bound (shown in blue).

**Figure 5:**
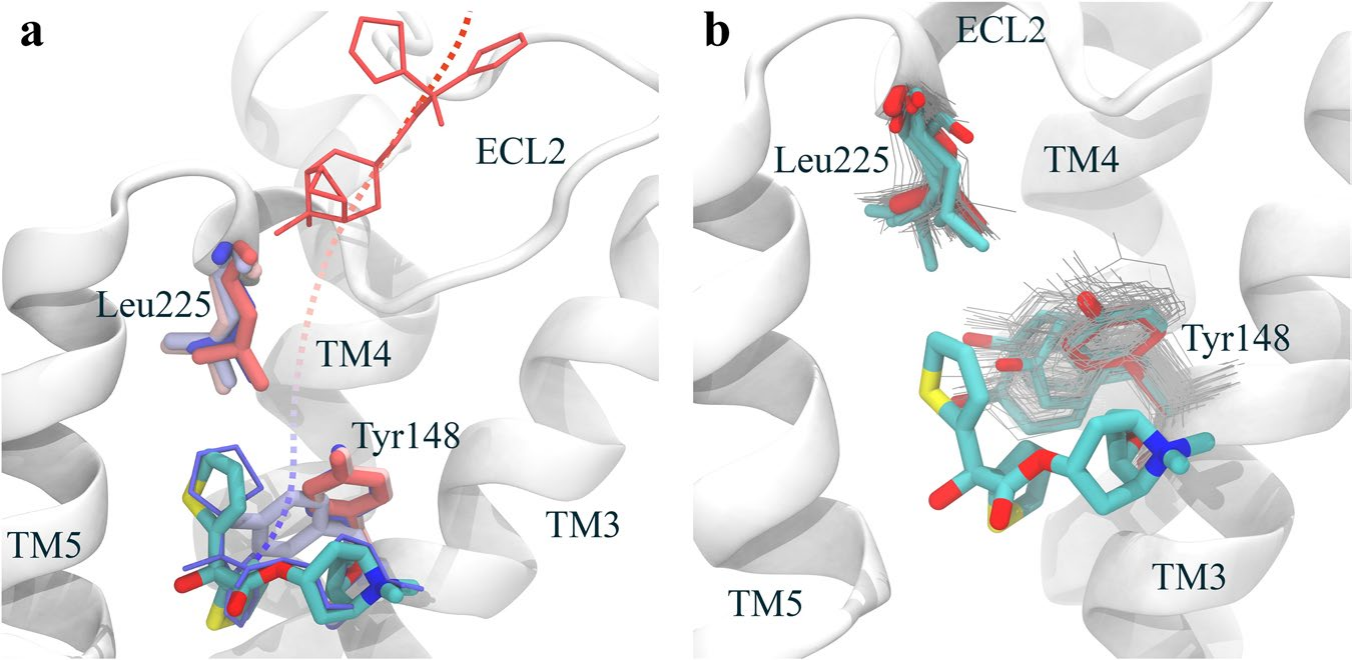
A-GPCR binding mechanism. (**a**) Different ligand snapshots showing the binding pathway from the initial structure (in red) to the bound pose (in blue), including Y148 and L225, which follow the same color-code. The white cartoon protein and the colored licorice ligand correspond to the bound crystal structure. **(b)** Side chain conformations for Y148 and L225, where the red licorice corresponds to the crystal structure. In grey lines, we show all the different conformations for those cluster centers along the adaptive process, and in colored licorice we show the resulting main conformations after a k-medoids clustering.

A-GPCR. GPCRs represent a great challenge for the modeling community. On top to the difficulties in obtaining atomistic models for these membrane proteins, we have the large plasticity of their extracellular domain (involved in ligand delivery and binding), and the buried nature of most of their binding sites. For A-GPCR, in particular, the extracellular loop 2 (ECL2) mobility has been reported to be involved in ligand binding, where a movement of L225 away from the orthosteric site permits a transient opening (rotation) of Y148 towards TM4, allowing tiotropium to bind, which closes again to form a lid in the binding pose^10^. As shown in Fig. 5a, in our simulations, we see a movement of L225 that is accompanied by a dihedral rotation of Y148 towards TM4, which allows binding. Once the ligand is bound, the tyrosine and the leucine move back to generate the binding pose. In Fig. 5b, we show the plasticity of these two residues, grouping all the involved cluster center side chain structures (in grey lines) into four main clusters using the k-medoids (in colored licorice) implemented in pyProCT^31^.

### Induced-Fit Docking

Predicting the non-biased binding mechanism is certainly a fancy computational effort, showing the capabilities of molecular modeling techniques. It aids in understanding the molecular mechanism of action, potentially finding, for example, alternative binding sites that might be used for rational inhibitor design. Another set of important simulations comprises docking refinement. Today, structure based design efforts ranging from virtual screening to fine tuning lead optimization activities, are hampered by having to properly handle the induced fit mechanisms. In this sense cross- and apo-docking studies, a significant less demanding modeling effort, constitute a better example. As seen in recent benchmark studies^28, 29, 32^ (or in the CSAR exercise^21^), standard PELE is possibly the fastest technique providing accurate answers in cross- and apo-docking, requiring on the order of 30–60 minutes wall clock time using ~16/32 trajectories in average.

**Figure 6:**
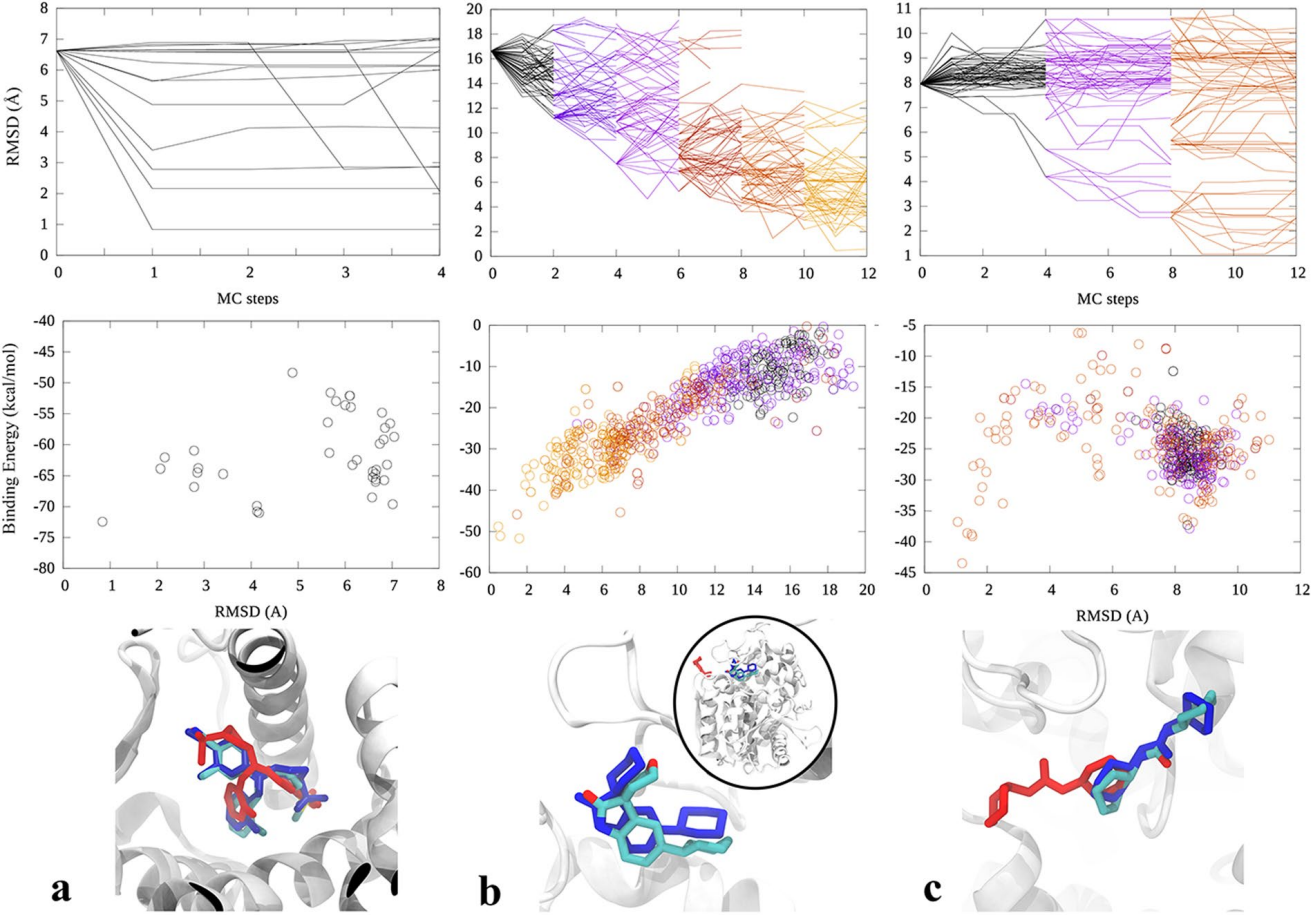
Induced-fit docking studies. (**a**) PR system: protein structure from PDB ID:1A28 and ligand structure from PDB ID:3KBA. (**b**) sHE system: protein structure from PDB ID:5AKE and ligand structure from PDB ID:5AM4. **(c)** sHE system: protein structure from PDB ID:5ALX and ligand structure from PDB ID:5AI5. In the upper panels we show the RMSD evolution along the simulation, in the middle ones the binding energy for the different RMSD values, and in the lower panels the native structure (atom-type colored), the lowest binding energy ligand structure (blue) and the starting ligand structure (red). Notice that in panel (b) the initial docking structure is slightly outside the active site (shown in the inset).

By introducing the adaptive sampling technique, we can now improve the simulation time to only few MC steps, as shown in Fig. 6, where we show the refinement of a wrong docked pose for the PR system and the application in cross docking for the soluble epoxide hydrolase (sEH), a tough benchmark system recently studied with standard PELE^32^ which requires significant active site reorganization. Notice that easy induced fit cases, such as PR requiring only a flip of the ligand, can be accomplished in one MC step, not representing any improvement from standard PELE. In difficult cases, such as for sEH, the adaptive scheme provides again significant improvement over standard simulations, shown in Supplementary Fig. 5. For example, notice in Supplementary Fig. 5a how standard PELE shows early non-productive low RMSD explorations (grey line achieving RMSD ~5 Å). This type of behavior motivated the development of the adaptive protocol.

Taking into account that the active site refinement MC steps require only 30 seconds (involving less protein perturbation and ligand translation, but more rotation), we can model the right pose in under 5 minutes using a modest computational cluster (32–64 processors), which allows refinement of a large number of docking poses or an interactive structural-guided optimization of a given lead.

## Discussion

Breakthrough advances in software and hardware are shifting the development of complex design processes to computer modeling. Still, accurately modeling the protein-ligand structure requires several hours of heavy computation, even when using special purpose machines or large clusters of processors. We have introduced here a new method, combining a reinforcement learning procedure with an all-atom molecular mechanics Monte Carlo technique, capable of providing non-biased accurate protein-ligand structures in minutes of CPU wall clock. This outstanding achievement opens the door for interactive usage, allowing to combine users’ expertise and intuition with *in silico* predictions.

A nice feature of adaptive-PELE is its scalability with computational resources; adding more computing cores (more trajectories) significantly reduces the wall clock computing time. While interactive refinement of active site poses requires only few processors, addressing the full binding mechanism (from solvent to the active site) requires significant more resources. While accessibility to cheap HPC will certainly increase in the near future, access to large computational resources for researchers is already a reality. Most pharmaceutical and biotech companies account for in-house large computational clusters, with several thousands of computing cores. Moreover, cloud-computing access is drastically increasing while reducing its cost; an hour of 128 computing cores sells today for ~5$ in *Amazon Cloud*. If associated security issues were a key negative aspect in the past, this has been largely solved: more and more companies have now developed cloud solutions (Schrödinger, Openeye, etc.)

In agreement with recent studies^1, 2, 5^ we show how all-atom molecular mechanics force fields are mature enough to sample and distinguish native like poses in complex protein-ligand systems, providing excellent means for elucidating the atomic detailed binding mechanism. Our tests involved difficult protein-ligand systems, including diverse and pharmacological relevant targets, such as the PR receptor, and a GPCR receptor for which extensive MD simulations could not provide a native like pose. While initial models were obtained from bound structures (not requiring large backbone reorganization), it constitutes a typical drug design modeling setup.

Overall, we have developed a computational breakthrough with remarkable performance in mapping the protein-ligand energy landscape, being able to reproduce the full binding mechanism in complex systems in less than half an hour, or the active site induced fit in less than 5 minutes. Being an initial implementation, future developments will seek further improvement, including: clustering based on protein-ligand atomic contact maps, alternative initial structures (within a cluster), different MAB strategies, etc. In addition, thermodynamics could be obtained by combining Adaptive-PELE with Markov state Models. While standard PELE already shows a competitive advantage as a sampling method^29, 32^, combining it with reinforcement learning techniques and high performance computing, provides a solid modeling technique to the drug-design community, with potential of being interactively used in computer aided drug design.

## Methods

### The Adaptive Algorithm

The algorithm is composed of three main steps: sampling, clustering, and spawning, which run in an iterative approach. In the sampling phase, a swarm of trajectories, in this paper in the range from tens to one thousand, are independently run. Conformations are then clustered, and the final spawning step chooses the seeds for the next iteration. By stopping simulations and adaptively spawning them, we circumvent the problem of getting trapped due to metastability, avoiding the waste of computational resources in oversampled regions.

#### Sampling

The sampling is usually the computational bottleneck of the process, so it is desired to use a method that can generate uncorrelated poses in a relatively short time. We chose PELE since it can introduce moderate conformational changes in few minutes, providing robust protein-ligand exploration, even for complex systems, within few hours of a mid-range computing cluster (~100 commodity computing cores)^18, 19, 33^. PELE is a two-stage MC algorithm that uses protein structure prediction procedures to generate proposals. In the first stage, the ligand is randomly moved, and the protein is perturbed using a normal mode analysis method based on an anisotropic network model (ANM)^17^. In the second one, the structure is relaxed with a side chain prediction and a minimization (with constraints on alpha carbons and the ligand center of mass), and the resulting proposal is accepted or rejected with the Metropolis criterion.

We use rounds (epochs) of *N* simulations (trajectories) of length *l*, each one running on a computing core (using an MPI implementation). A larger *N* is expected to reduce the wall-clock time to see binding events, whereas *l* should be as small as possible to exploit the communication between explorers but long enough for new conformations to advance in the landscape exploration. While we use PELE in this work, one could use different sampling programs such as MD as well.

#### Clustering

We used the leader algorithm^34^ based on the ligand RMSD, where each cluster has a central structure and a similarity RMSD threshold, so that a structure is said to belong to a cluster when its RMSD with the central structure is smaller than the threshold. The process is speeded up using the centroid distance as a lower bound for the RMSD (see Supplementary Information). When a structure does not belong to any existing cluster, it creates a new one being, in addition, the new cluster center. In the clustering process, the maximum number of comparisons is *k*·*n*, where *k* is the number of clusters, and *n* is the number of explored conformations in the current epoch, which ensures scalability upon increasing number of epochs and clusters.

We assume that the ruggedness of the energy landscape grows with the number of protein-ligand contacts, so we make RMSD thresholds to decrease with them, ensuring a suitable discretization in regions that are more difficult to sample. This concentrates the sampling in interesting areas, and speeds up the clustering, as fewer clusters are built in the bulk.

#### Spawning

In this phase, we select the seeding (initial) structures for the next sampling iteration with the goal of improving the search in poorly sampled regions, or to optimize a user-defined metric; the emphasis in one or another will motivate the selection of the spawning strategy. Naively following the path that optimizes a quantity (e.g. starting simulations from the structure with the lowest SASA or best interaction energy) is not a sound choice, since it will easily lead to cul-de-sacs. Using MAB as a framework, we implemented different schemes and reward functions, and analyzed two of them to understand the effect of a simple diffusive exploration in opposition to a semi-guided one.

The first one, namely inversely proportional, aims to increase the knowledge of poorly sampled regions, especially if they are potentially metastable. Clusters are assigned a reward, *r*:

1 $$r=\frac{\rho }{C}$$

where *ρ*, is a designated density and C is the number of times it has been visited. We choose *ρ* according to the ratio of protein-ligand contacts, again assumed as a measure of possible metastability, aiming to ensure sufficient sampling in the regions that are harder to simulate. The 1/C factor guarantees that the ratio of populations between any two pairs of clusters tends to the ratio of densities in the long run (one if densities are equal). The number of trajectories that seed from a cluster is chosen to be proportional to its reward function, *i.e*. to the probability to be the best one, which is known as the Thompson sampling strategy^35, 36^. The procedure generates a metric-independent diffusion.

The second strategy is a variant of the well-studied *ε*-greedy^25^, where a 1−*ε* fraction of explorers are using Thompson sampling with a metric, *m*, that we want to optimize, and the rest follow the inversely proportional scheme. Metrics are typically used in PELE to extract information and to drive the system towards some determined actions. They include, for example, the binding energy, the SASA of the ligand, distances between atoms, etc. Depending on whether we want to maximize or minimize *m*, *r* is respectively defined as:

2 $${r}_{i}={m}_{i,{\rm{\min }}}-{m}_{{\rm{\min }}}$$

3 $${r}_{i}={m}_{{\rm{\max }}}-{m}_{i,{\rm{\max }}},$$

where m_i,max_ and m_i,min_ are the maximum and minimum metric values within the *i*-th cluster respectively, and *m*_*min*_ and *m*_*max*_ are the overall metric minimum and maximum.

The adaptive python code is public on GitHub: https://github.com/AdaptivePELE/AdaptivePELE

### Benchmark Systems

We have chosen four systems with different levels of complexity: the trypsin-benzamidine, the PR nuclear hormone receptor with its endogenous ligand and two different GPCRs with a potent inverse agonist and an antagonist ligand respectively; these last three systems represent current pharmaceutical targets, allowing us to evaluate the viability of the protocol in real drug design processes.

The binding of trypsin with benzamidine (PDB ID: 3PTB) has been widely used as a benchmark system^6, 37, 38^. It is the smallest and least flexible receptor and ligand, being the system that requires the least computational time.

PR with its endogenous ligand (PDB ID: 1A28) belongs to the family of nuclear hormone receptors (NHR) and is an important pharmaceutical target. NHRs have been recently studied combining crystallography and PELE^19^, including studies with PR^30^, where it was found that protein plasticity was crucial for the ligand to enter the active site.

We also tested two different GPCRs with two different ligands, tiotropium (PDB ID: 4DAJ) and CP-376395 (PDB ID: 4K5Y). GPCRs are a class of transmembrane proteins involved in the signaling of a wide range of biological functions and key pharmaceutical targets. 4DAJ is an M3 muscarinic acetylcholine receptor belonging to class A GPCRs, for which extensive MD simulations have already been performed. Despite the use of the Anton supercomputer and of 16 μs of MD production time^10^, binding of tiotropium, a bronchodilator drug, into the orthosteric site could not be reported, only seeing binding to an extracellular site vestibule. 4K5Y is a class B GPCR, involved in the treatment of anxiety and depression, whose bent transmembrane helix (TM) 7 produces a pronounced V-shape allowing the ligand to enter deeper into the channel^39^. While no binding simulations have been reported to our knowledge, the conformational changes between the apo and the holo structures have been recently studied running 100 ns MD simulations, with and without the antagonist ligand^40^. In addition, binding dissociation pathways have been studied with random acceleration molecular dynamics^41^.

### Setup

#### System preparation

In order to test the potential of the new methodology in exploring the binding mechanism, we started simulations with a model where the ligand is placed 20 Å from the bound pose (see Fig. 1), and constrained its movements to a sphere of 15 Å, the center of which was placed in the middle point between the native and initial configurations. Structures were prepared with Schrödinger’s Protein Wizard^42^. Simulations were run with the OPLS2005 force field and the OBC implicit solvent^43^. Ligands’ atomic charges were parameterized with RESP quantum charges, obtained with Jaguar^44^ optimizations at the DFT-B3LYP and 6–31 G** + level of theory.

#### PELE control file

The same parameters were used for both adaptive and non-adaptive runs. The ligand translation was set to be dependent on its (relative) solvent accessible surface area (SASA), being 3 Å for SASA > 0.6 whereas it otherwise ranged randomly from 0.75 to 1.5 Å in the protein vicinity; the translation direction was kept for four consecutive steps. Ligand rotation was randomly set between 20° and 60°. For the protein backbone perturbation, performed with a probability of 0.25, the lowest six ANM normal modes were randomly mixed with a maximum displacement of 1.5 Å. The same PELE control file has been used for all systems with except for the alpha carbon constraints in the relaxation step: since it was reported that the lipid bilayer was found not to play a significant role in the binding in the GPCR^40^, we speeded up simulations removing the membrane and adding constraints of 5 kcal/mol/Å^2^ every 10-th alpha carbons in the TMs, setting it to 0.2 kcal/mol/Å^2^ in TRP and PR.

#### Algorithm parameters

Although a general set of parameters has been optimized and used in this work, users are encouraged to change them; limiting factors to consider are discussed in this section.

In the sampling phase, we use exploration rounds of *l* = 4 steps, which ensures epochs of less than four minutes with the current Marenostrum 3 processors at the Barcelona Supercomputing Center (SandyBridge-EP 2.6 GHz processors). Protein conformational changes can already be captured with four steps, and longer simulations were leading to poorer performance.

The number of protein-ligand contacts is used as a measure of the sampling complexity, as more contacts lead to more competing interactions and, thus, more energy barriers and metastability. We consider that a pair of protein (alpha carbons only) and ligand atoms are in contact if their distance is less than 8 Å, following ref. 23. In our implementation, we use as a parameter the ratio of the number of contacts per ligand heavy atom, *c*, since it is less system dependent, and regard those conformations with *c* > 1 as difficult to sample, which correspond to poses in the protein vicinity, and those with *c* < = 0.5 as easy, which correspond to largely solvent exposed poses.

We tried three different combinations of cluster threshold and density values, and summarized in the table of Supplementary Fig. 6. Clusters need to be small enough so that one can distinguish (relevant) different conformations. We select the thresholds with a function composed of linearly decreasing step functions in *c*, from 5 Å in the solvent (*c* < = 0.5) to 2 Å in the protein frame (*c* > 1). This ensures sufficient discretization in those regions that are difficult to sample, not spending too many resources in the bulk (Supplementary Fig. 6a). Using the same threshold everywhere, requires significant more sampling to reach native like poses (Supplementary Fig. 6b), since it introduces 3 times more clusters (Supplementary Fig. 6d).

In the spawning, the density value is chosen inversely proportional to the cluster volume (1/V). We tried different density functions. For example, $\rho$ = 1 allows seeing binding events, but it divides exploration efforts in the whole domain, as can be seen in (Supplementary Fig. 6c).

## Electronic supplementary material

Supplementary Information
