# Supplementary material for: Adaptive simulations, towards interactive protein-ligand modeling
Source: Sci Rep. 2017 Aug 16;7:8466. doi: 10.1038/s41598-017-08445-5 (PMC5559483; doi:10.1038/s41598-017-08445-5)
Supplement: Supplementary file 1 — Supplementary Information [file 41598_2017_8445_MOESM1_ESM.pdf]

Adaptive simulations, towards interactive protein-ligand modeling

Daniel Lecina[1], Joan Francesc Gilabert[1] and Victor Guallar[1,2]

[1]Barcelona Supercomputing Center, Joint BSC-CRG-IRB Research Program in Computational Biology, Jordi Girona 29, E-08034 Barcelona, Spain
[2]ICREA, Passeig Lluís Companys 23, E-08010 Barcelona, Spain

Supplementary Information
Supplementary information includes: i) methodological clustering details; ii) Clustering exploration results (Fig. 1); iii) Energy landscape exploration of TRP, A-GPCR and PR (Figs 2-4); iv) Complete table with all binding time statistics; v) Standard PELE induced fit simulations on sEH; vi) Clustering parameters configurations (Fig. 5)

Centroid distance as a lower bound for the RMSD.

In the clustering, we have a ligand structure that we want to cluster, and a cluster center (the reference structure) with coordinates $\mathbf{r}$ and $\mathbf{r}_{\mathrm{REF}}$, respectively.
The distance vector between the *i*-th atom in both structures is defined as:

$$\boldsymbol{d}_i(\boldsymbol{r}_i,\boldsymbol{r}_{\mathrm{REF},i}) = \boldsymbol{r}_i - \boldsymbol{r}_{\mathrm{REF},i} \quad (1)$$

For the sake of brevity, we will avoid recalling explicitly the dependence on the two sets of coordinates: $\boldsymbol{d}_i \equiv \boldsymbol{d}_i(\boldsymbol{r}_i,\boldsymbol{r}_{\mathrm{REF},\,i})$. The distance between a pair of atoms corresponds to its modulus: $d_i = ||\boldsymbol{d}_i||$.
The centroid distance, c_d, between both structures is:

$$c_d(\boldsymbol{d}_i) = ||\sum_i \frac{\boldsymbol{d}_i}{N}|| \quad (2)$$

where the summation extends over all N ligand atoms.

After superposing the protein alpha carbons, the ligand RMSD is calculated with:

$$\mathrm{RMSD}\,(d_i) = \sqrt{\sum_i \frac{{d_i}^2}{N}} \quad (3)$$

where the summation again extends over all ligand atoms.

The Cauchy-Schwarz inequality states that in an n-dimensional Euclidean space: $|\langle \boldsymbol{u} \cdot \boldsymbol{v} \rangle| \leq ||\boldsymbol{u}|| \cdot ||\boldsymbol{v}||$, where $\langle .,. \rangle$ is the inner product. Applying it to **u** and **v** such that $u_i = \frac{d_i}{N}$ and $v_i = 1, \forall i \mid i \in [1, N]$:

$$\sum_i \frac{d_i}{N} \leq \sqrt{\sum_i \left(\frac{d_i}{N}\right)^2 \cdot N} = \sqrt{\sum_i \frac{d_i^2}{N}} = \mathrm{RMSD}(d_i) \quad (4)$$

Using the triangle inequality for $\mathbf{d}_i$: $\left||\sum_i \boldsymbol{d}_i|\right| \leq \sum_i \left||\boldsymbol{d}_i|\right| = \sum_i d_i$, and dividing it by N, we obtain:

$$c_d(\boldsymbol{d}_i) = ||\sum_i \frac{\boldsymbol{d}_i}{N}|| \leq \sum_i \frac{d_i}{N} \tag{5}$$

Combining Eq. (4) and Eq. (5) we obtain:

$$c_d(\boldsymbol{d}_i) \leq \mathrm{RMSD}(d_i), \tag{6}$$

as we wanted to prove. Intuitively, the equality applies when the structure is a translation of the reference structure, and the inequality applies when there is a translation and rotation.

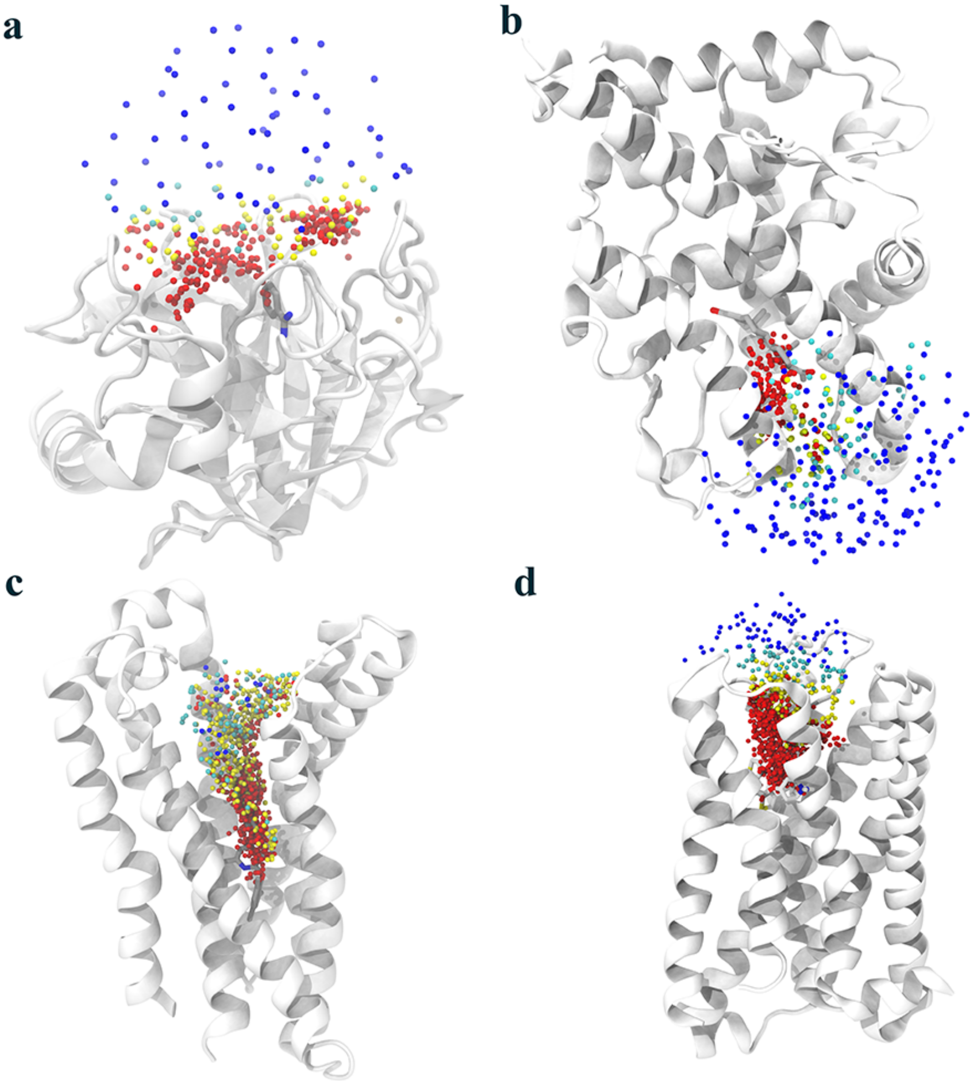

Supplementary Figure 1 | Cluster exploration in a typical binding simulation with 1024 trajectories. In white, we show the native structure, and each cluster is marked with the ligand's center of mass with a color that represents its number of contacts and RMSD threshold: in blue those with $c <= 0.5$ and a threshold of 5Å, in cyan those with $0.5 < c <= 0.75$ and a threshold of 4Å, in yellow those with $0.75 < c <= 1$ and a threshold of 3Å, and in red, those with $c > 1$ and a threshold of 2Å. Panel **(a)** corresponds to TRP, panel **(b)** to PR, panel **(c)** to B-GPCR and panel **(d)** to A-GPCR.

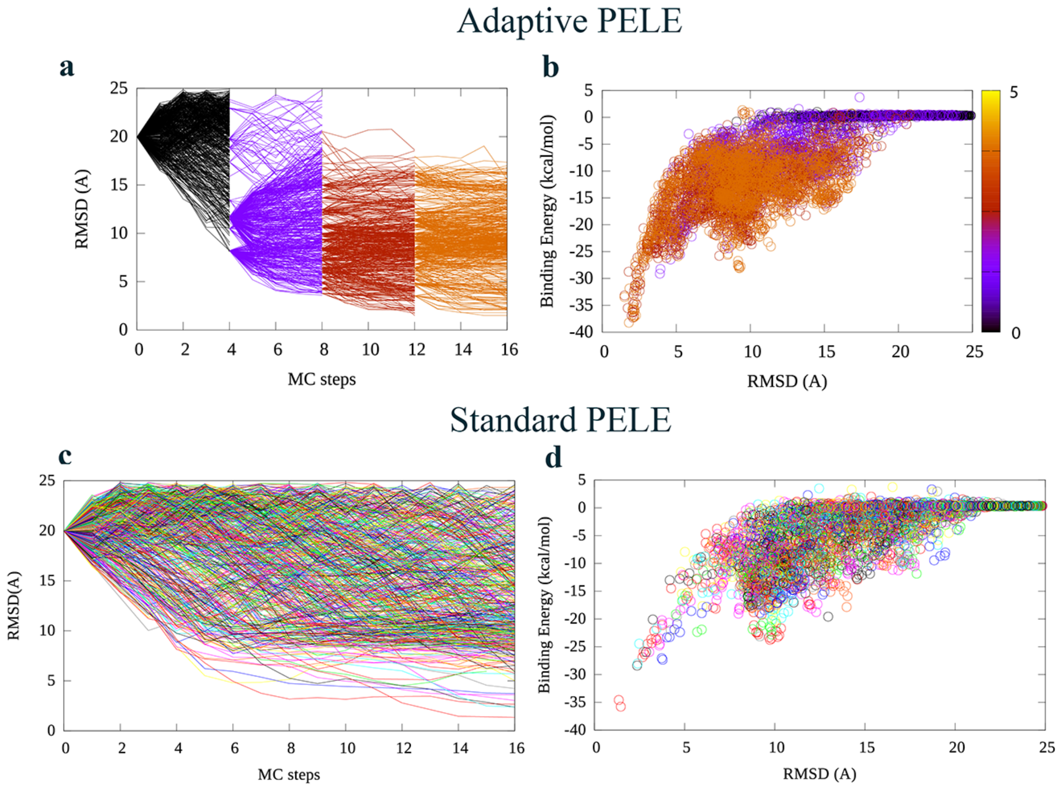

Supplementary Figure 2 | Energy landscape exploration of TRP with 512 different explorers. (a,b) The RMSD variation along MC steps and the binding energy against the RMSD for the adaptive results. Each color code corresponds to a different epoch number, for a total of 4 adaptive iterations. **(c,d)** Analogous plots for the standard executions. Each color corresponds to a different trajectory (performed in a different computing core).

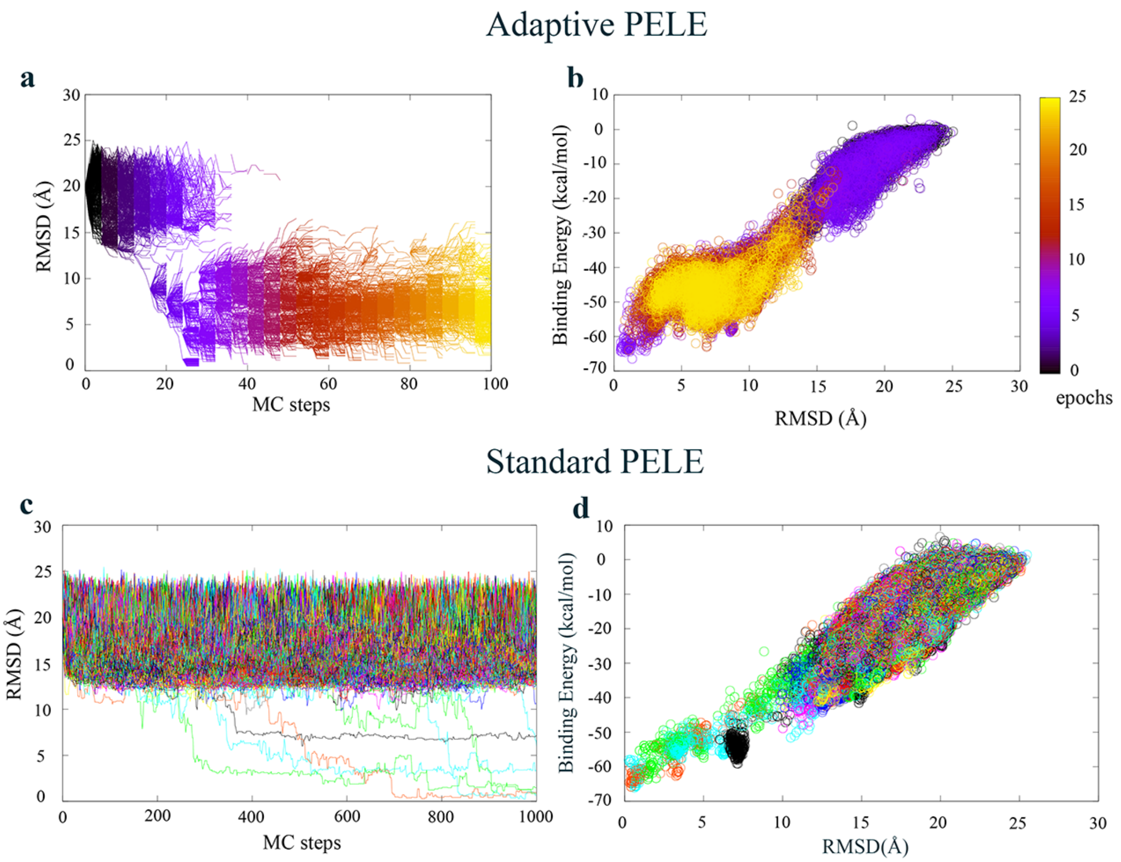

Supplementary Figure 3 | Energy landscape exploration of PR with 512 different explorers. (a,b) The RMSD variation along MC steps and the binding energy against the RMSD for the adaptive results. Each color code corresponds to a different epoch number, for a total of 24 adaptive iterations. **(c,d)** Analogous plots for the standard executions. Each color corresponds to a different trajectory (performed in a different computing core).

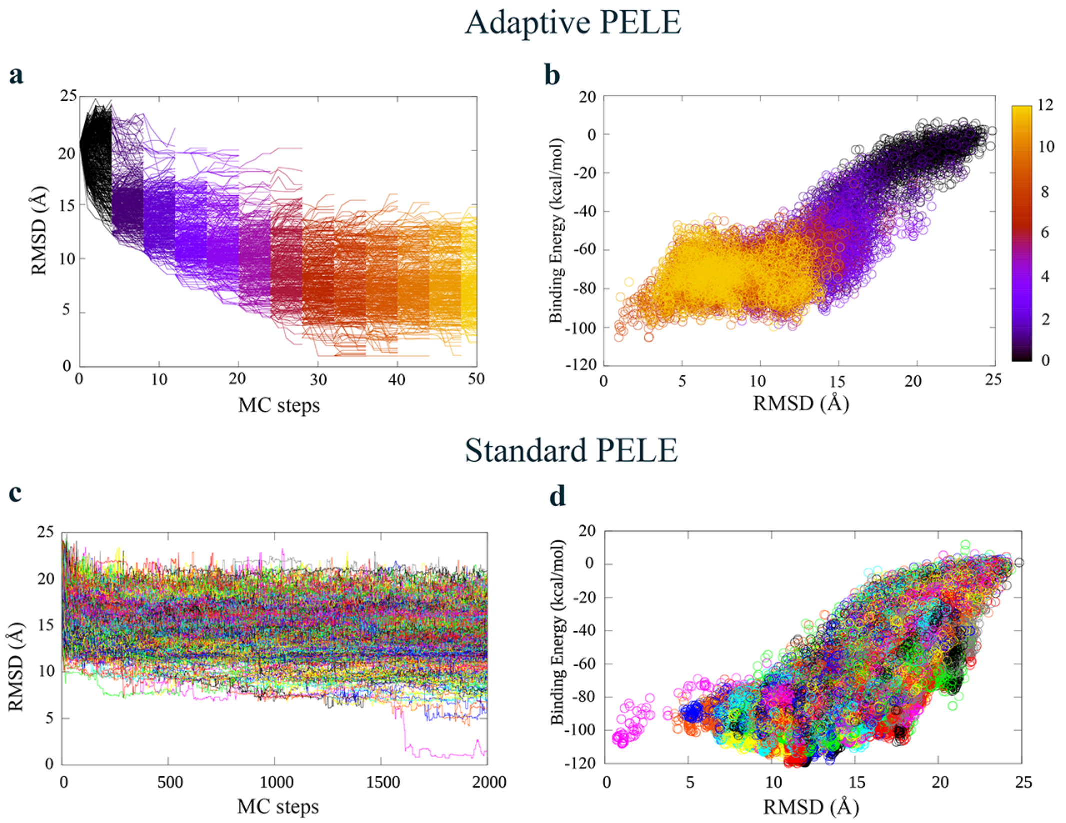

Supplementary Figure 4 | Energy landscape exploration of A-GPCR with 512 different explorers. (a,b) The RMSD variation along MC steps and the binding energy against the RMSD for the adaptive results. Each color code corresponds to a different epoch number, for a total of 12 adaptive iterations. **(c,d)** Analogous plots for the standard executions. Each color corresponds to a different trajectory (performed in a different computing core).

	32	64	128	256	512	1024
			TRP			
🔴	39±30	26±10	18±7	14±4	11±3	11±2
🔵	26±20	21±9	12±3	9±2	10±2	9±1
🟢	19±7	13±3	10±2	8±2	9±1	8±1
🟠	19±9	13±4	10±2	9±3	9±2	8±2
			PR			
🔴	-	1830±1250	1590±1150	1510±930	610±300	500±310
🔵	460±270	160±90	160±130	110±60	42±20	30±9
🟢	270±160	160±50	120±90	73±40	56±40	32±6
🟠	230±110	200±90	97±60	65±30	43±10	33±10
			B-GPCR			
🔴	2200±1000	1100±800	740±300	490±200	360±130	350±100
🔵	420±500	140±50	87±20	59±20	39±10	36±10
🟢	200±90	130±60	82±40	55±10	48±10	35±10
🟠	110±40	98±30	67±20	53±7	40±6	33±8
			A-GPCR			
🔴	-	-	2440±700	1260±830	1230±640	910±460
🔵	200±100	115±40	56±10	45±10	30±4	25±4
🟢	230±100	76±22	74±30	45±10	30±5	23±4
🟠	110±50	85±30	53±20	42±10	32±3	25±3

🔴 Std. PELE, 🔵 : Inversely Proportional, 🟢 B.E. ε-greedy, 🟠 RMSD ε-greedy

Supplementary Table 1 | Binding times for all studied systems and strategies. Results show the MC steps averaged over ten independent runs. For PR with 32 processors and A-GPCR for 32 and 64, we did not observe any binding event in more than half of the runs. The color code corresponds to the strategy; red for non-adaptive PELE, blue for the inversely proportional strategy, green for the binding energy ε-greedy and orange for the RMSD ε-greedy.

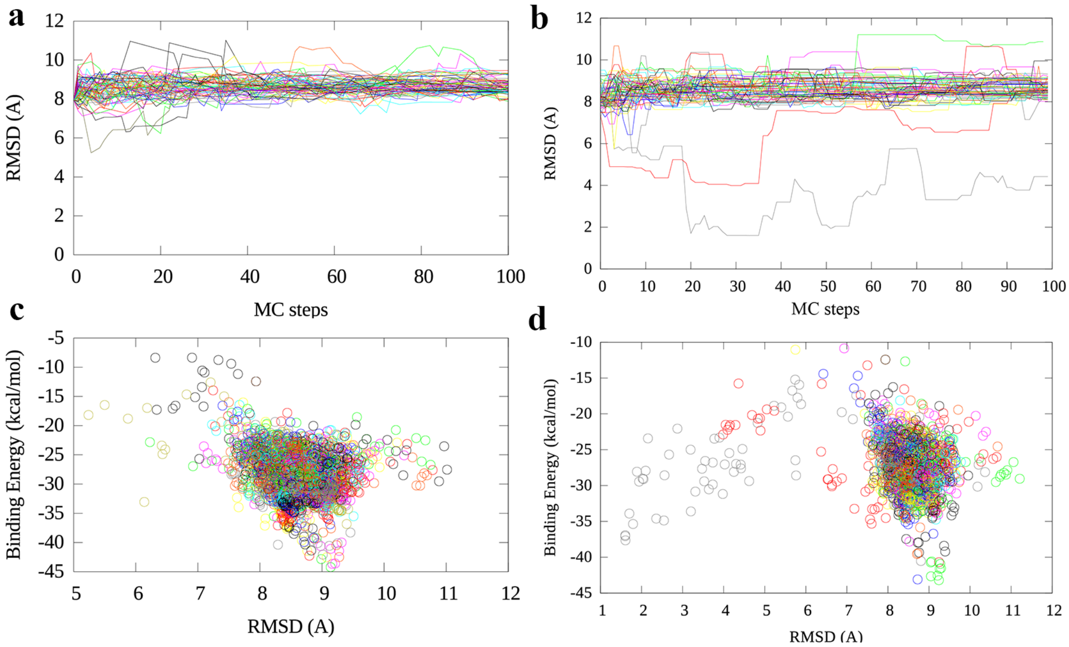

Supplementary Figure 5 | Standard PELE induced-fit docking studies. Two different cross docking simulations with 64 trajectories are shown for the sHE system: protein structure from PDB ID:5ALX and ligand structure from PDB ID:5AI5 **(a,b)** Evolution of the ligand RMSD to the bound crystal along the simulation. **(c,d)** Evolution of the binding energy for the different RMSD values.

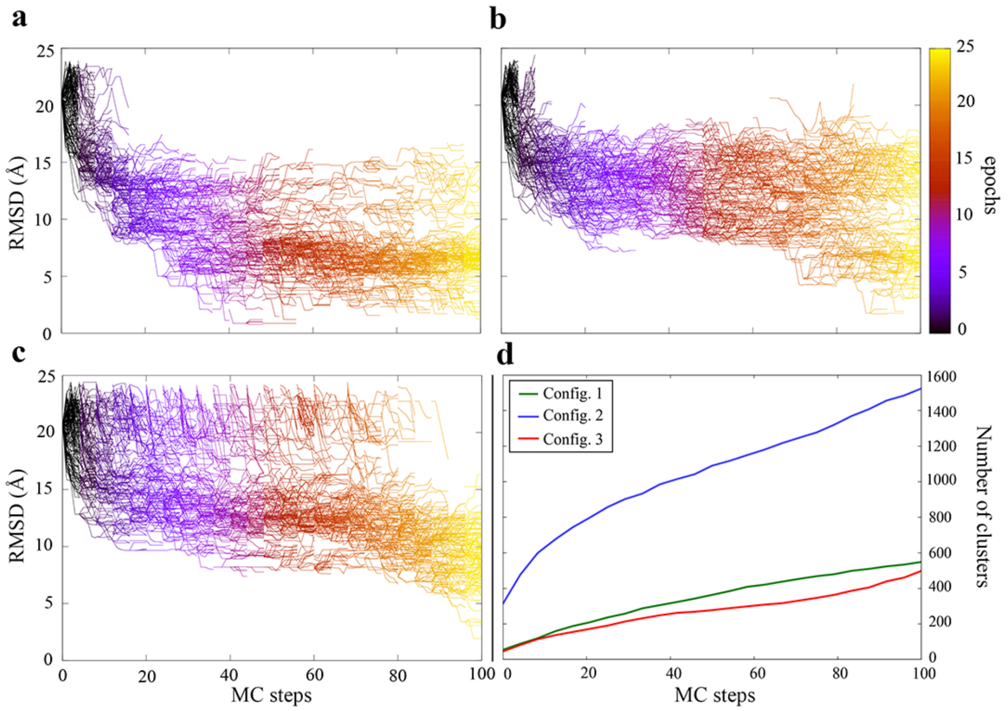

	Density	RMSD thresholds
Configuration 1	∝ 1/V	Linearly decreasing
Configuration 2	∝ 1/V	Constant (2Å)
Configuration 3	Constant	Linearly decreasing

Supplementary Figure 6 | Clustering parameters configurations. (a,b,c) Evolution of the ligand RMSD to the bound crystal along the simulation, corresponding to configuration 1, *i.e.* the one that is used throughout the paper, configuration 2 and 3, respectively. **(d)** Evolution of the number of clusters. Those results correspond to A-GPCR using 128 processors and 100 MC steps for three different parameter configurations.
